# Supplementary material for: Detection of senescence using machine learning algorithms based on nuclear features
Source: Nat Commun. 2024 Feb 3;15:1041. doi: 10.1038/s41467-024-45421-w (PMC10838307; doi:10.1038/s41467-024-45421-w)
Supplement: Supplementary file 1 — Supplementary Information [file 41467_2024_45421_MOESM1_ESM.pdf]

Supplementary Information

Detection of senescence using machine learning algorithms based on nuclear features

Imanol Duran[1,2], Joaquim Pombo[1,2], Bin Sun[1,2], Suchira Gallage[1,2,3,4], Hiromi Kudo[5], Domhnall McHugh[1,2], Laura Bousset[1,2], Jose Efren Barragan Avila[3], Roberta Forlano[6], Pinelopi Manousou[6], Mathias Heikenwalder[3,4,7], Dominic J. Withers[1,2], Santiago Vernia[1,2], Robert D. Goldin[5], and Jesús Gil[1,2,*]

[1]MRC Laboratory of Medical Sciences (LMS), Du Cane Road, London, W12 0NN, United Kingdom.

[2]Institute of Clinical Sciences (ICS), Faculty of Medicine, Imperial College London, Du Cane Road, London W12 0NN, United Kingdom.

[3]Division of Chronic Inflammation and Cancer, German Cancer Research Center (DKFZ), Heidelberg, Im Neuenheimer Feld 280, 69120 Heidelberg, Germany.

[4]M3 Research Center for Malignome, Metabolome and Microbiome, Faculty of Medicine, University of Tuebingen, Otfried-Müller-Straße 37,72076 Tübingen, Germany.

[5]Section for Pathology, Division of Digestive Diseases, Department of Metabolism, Digestion and Reproduction, Faculty of Medicine, Imperial College London, London W21NY, United Kingdom.

[6]Liver Unit, Section of Hepatology and Gastroenterology, Division of Digestive Diseases, Department of Metabolism, Digestion and Reproduction, Faculty of Medicine, Imperial College London, London W21NY, United Kingdom.

[7]Cluster of Excellence iFIT (EXC 2180), Eberhard Karls University, Tübingen, Germany.

***Corresponding author:** jesus.gil@imperial.ac.uk

Including:

- 16 Supplementary Figures.
- 5 Supplementary Tables.

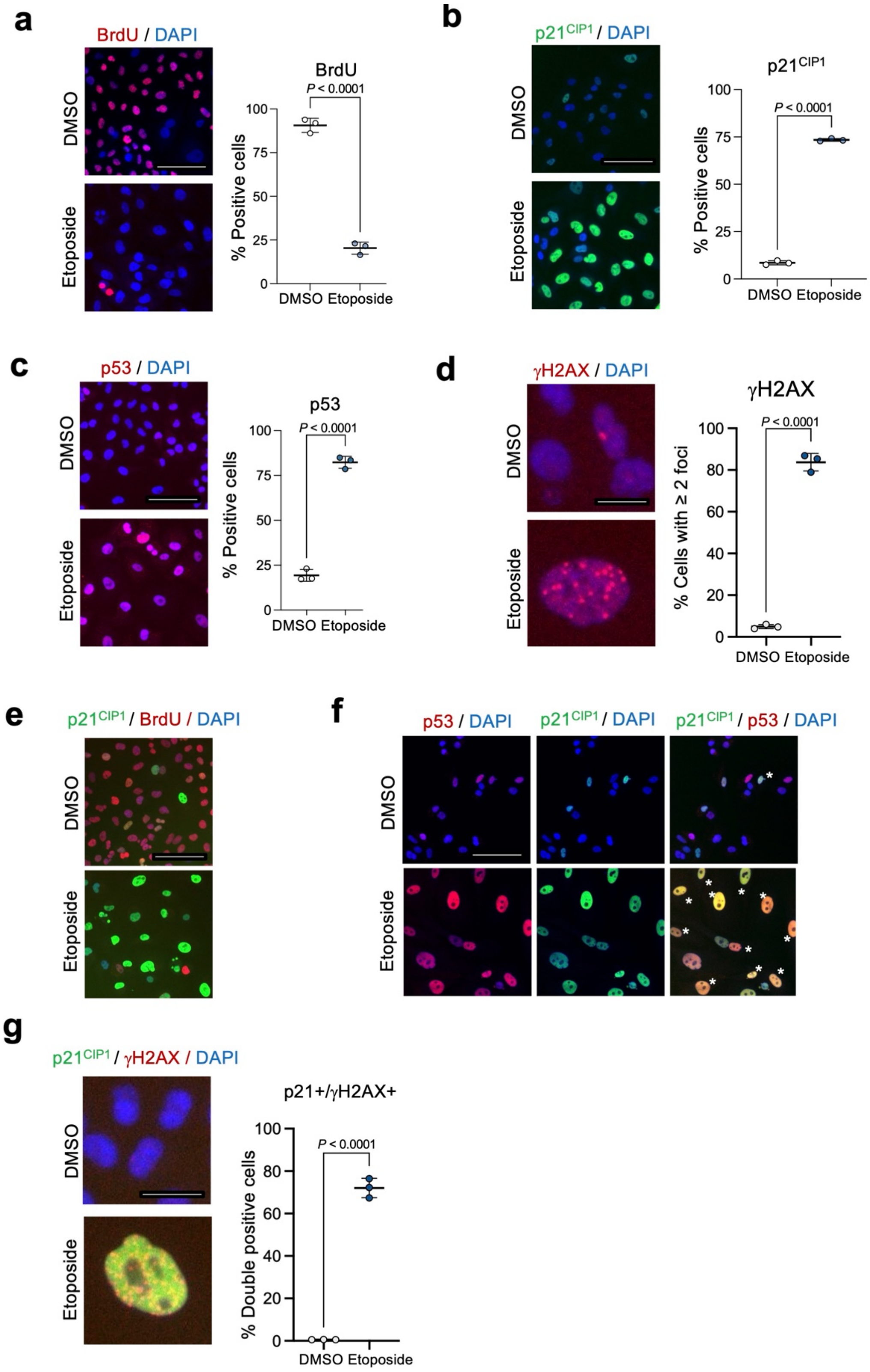

Supplemental Figure 1. Characterisation of senescence induced in A549 cells treated with etoposide. a-d, Quantification (right) and representative images (left) of cellular senescence markers assessed by immunofluorescence. BrdU incorporation (**a**), p21^{CIP1} (**b**), p53 (**c**), and γH2AX (**d**). (n=3). **e-f**, Representative images of

$p21^{Cip1}$/BrdU double staining (**e**) and $p21^{Cip1}$/p53 double staining (**f**) in A549 cells 7 days after DMSO or etoposide treatment. $p21^{Cip1}$/p53 double positive cells are marked by white asterisks (*). **g**, Quantification (right) of cells double positive for $p21^{CIP1}$and γH2AX (n=3). Representative images are shown on the left. Scale bars: 100μm (a, b, c, e, f) and 20μm (d, g). Statistical significance was calculated using unpaired, two-tailed, Student's *t*-tests. All measures represent the mean with s.d. n represents the number of replicates. Source Data are provided in the Source Data File.

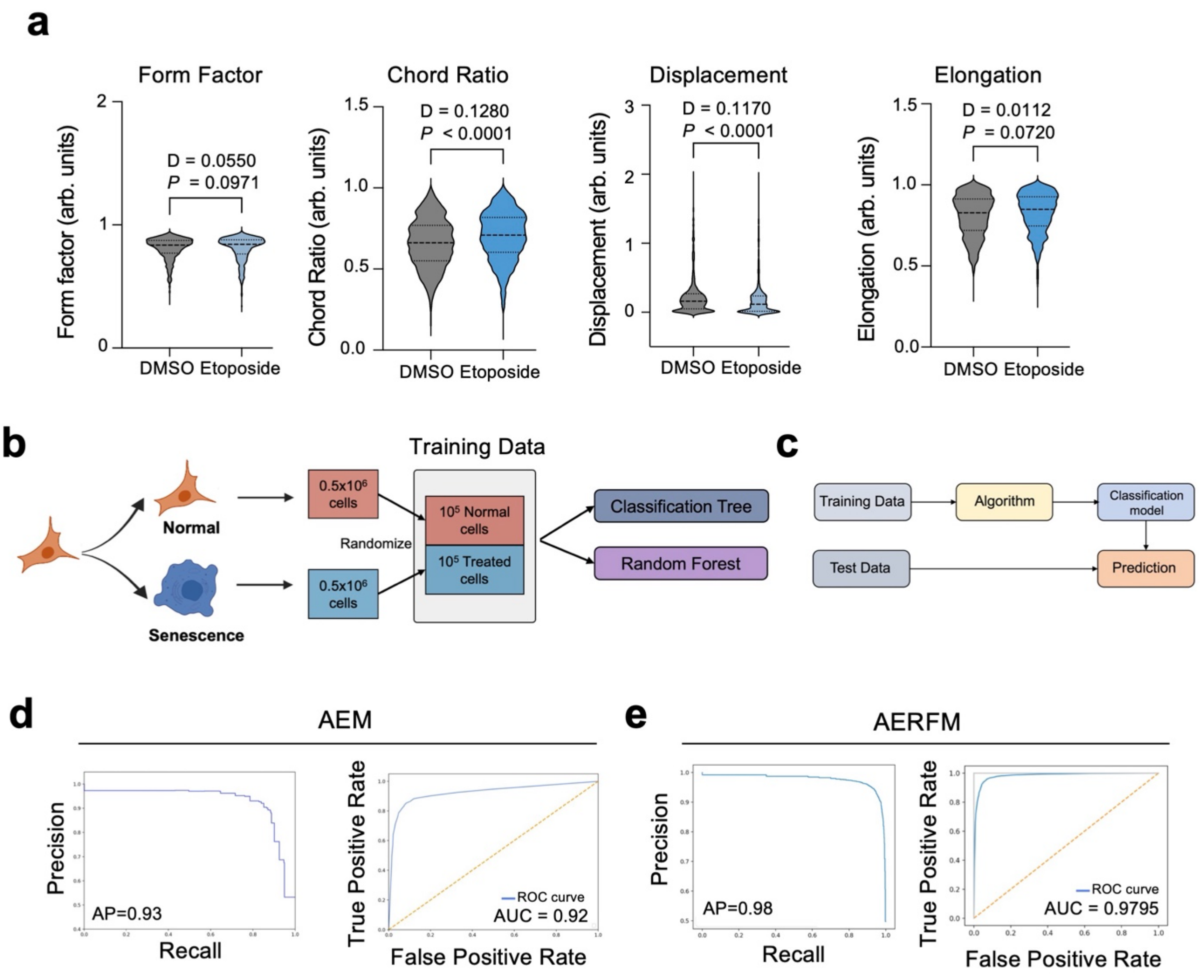

Supplemental Figure 2. Nuclear features can be used to identify senescent cells. **a**, Quantification of different nuclear morphological features in DMSO and etoposide-treated A549 cells 7 days post-treatment (n=1,000 cells per group). Data of a representative experiment out of 3. Dash lines in violin plots represent the median values and the pointed lines represent the quartiles. Kolmogorov-Smirnov test was performed to assess probability distribution, with D value indicated. **b**, Experimental design for the development of senescence-detecting algorithms. **c**, Workflow for developing senescence-detecting algorithms and validation process. **d-e**, Precision-recall curve (left) and area under the curve (AUC, right) for the analysis of training data using the A549 etoposide model (AEM, **d**) or A549 etoposide random forest model (AERFM, **e**) senescence classifiers. Average Precision (AP) was calculated using the area under the curve (AUC) of the Precision-Recall curve (left) while the relationship between true positive and false positive rates was calculated with their corresponding AUC (right). Source Data are provided in the Source Data File.

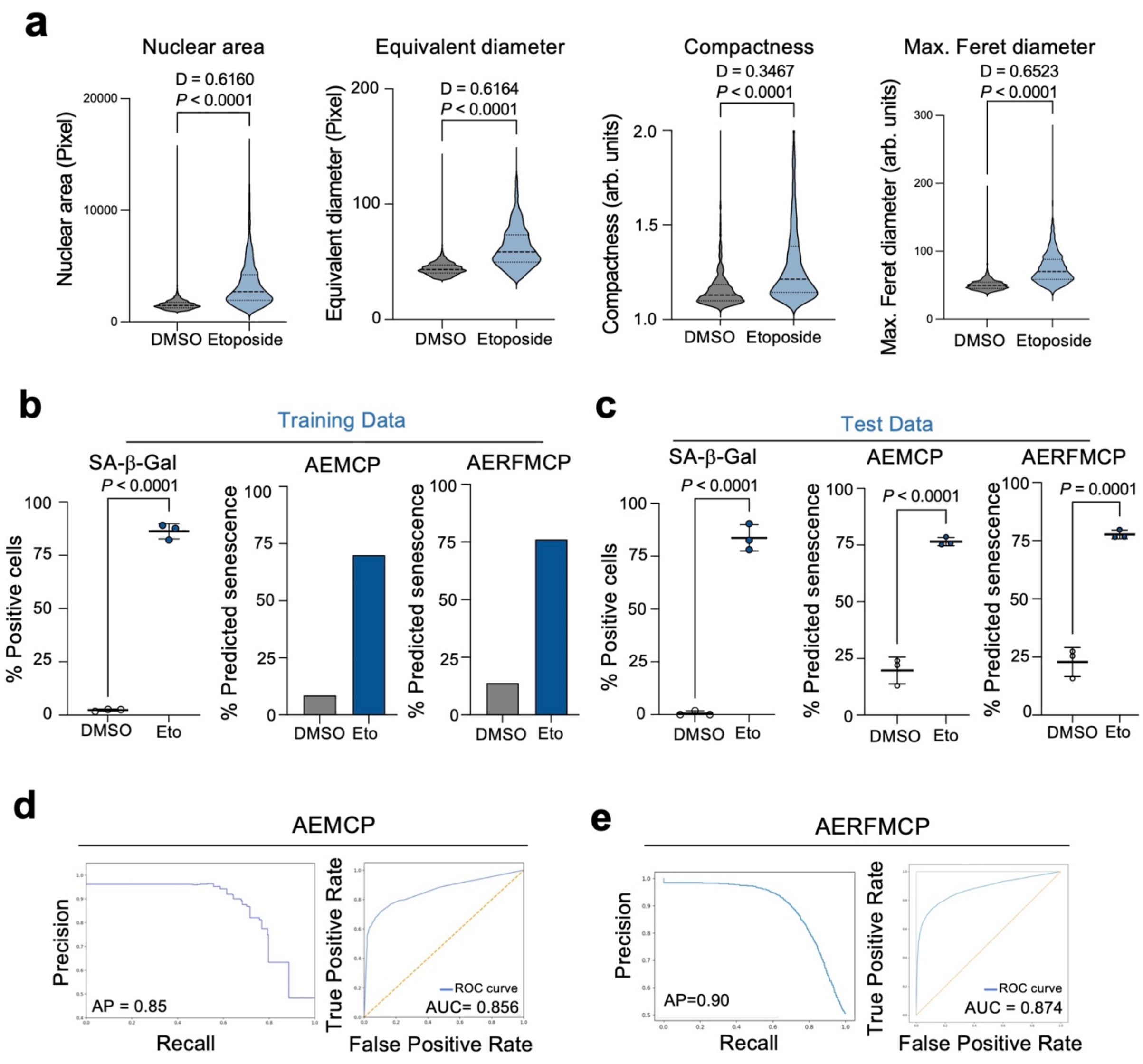

Supplemental Figure 3. Devising senescence classifiers using CellProfiler data. **a**, Quantification of nuclear features for DMSO and etoposide-treated A549 cells 7 days post-treatment (n=1,000 cells per group). Data of a representative experiment out of 3. Dash lines represent median and quartile values. Kolmogorov-Smirnov test was performed to assess probability distribution, with D value indicated. **b**, Analysis of training datasets of A549 cells treated with DMSO or etoposide. Percentage of SA-β-galactosidase (SA-β-Gal) positive cells (left, n=3) and percentage of predicted senescent cells using the A549 etoposide Cell Profiler model (AEMCP, centre) and A549 etoposide random forest Cell Profiler (AERFMCP, right) senescence classifiers. **c**, Analysis of test datasets of A549 cells treated with DMSO or etoposide. Percentage of SA-β-Gal positive cells in DMSO (normal) and etoposide-treated A549 cells (left, n=3). Percentage of predicted senescent cells in the validation datasets using the AEMCP (middle) and AERFMCP (right) classifiers. **d-e**, Precision-recall curve (left)

and area under the curve (AUC, right) for the analysis of test data using the AEMCP (**d**) or AERFMCP (**e**) senescence classifiers. Average precision (AP) was calculated using the area under the curve (AUC) of the precision-recall curve (left). The relationship between true positive and false positive rates was calculated with Its corresponding AUC (right). Statistical significance in **a**, **b,** and **c** was calculated using unpaired, two-tailed, Student's *t*-tests. Data represent mean ±s.d. n represents the number of replicates except indicated otherwise. Source Data are provided in the Source Data File.

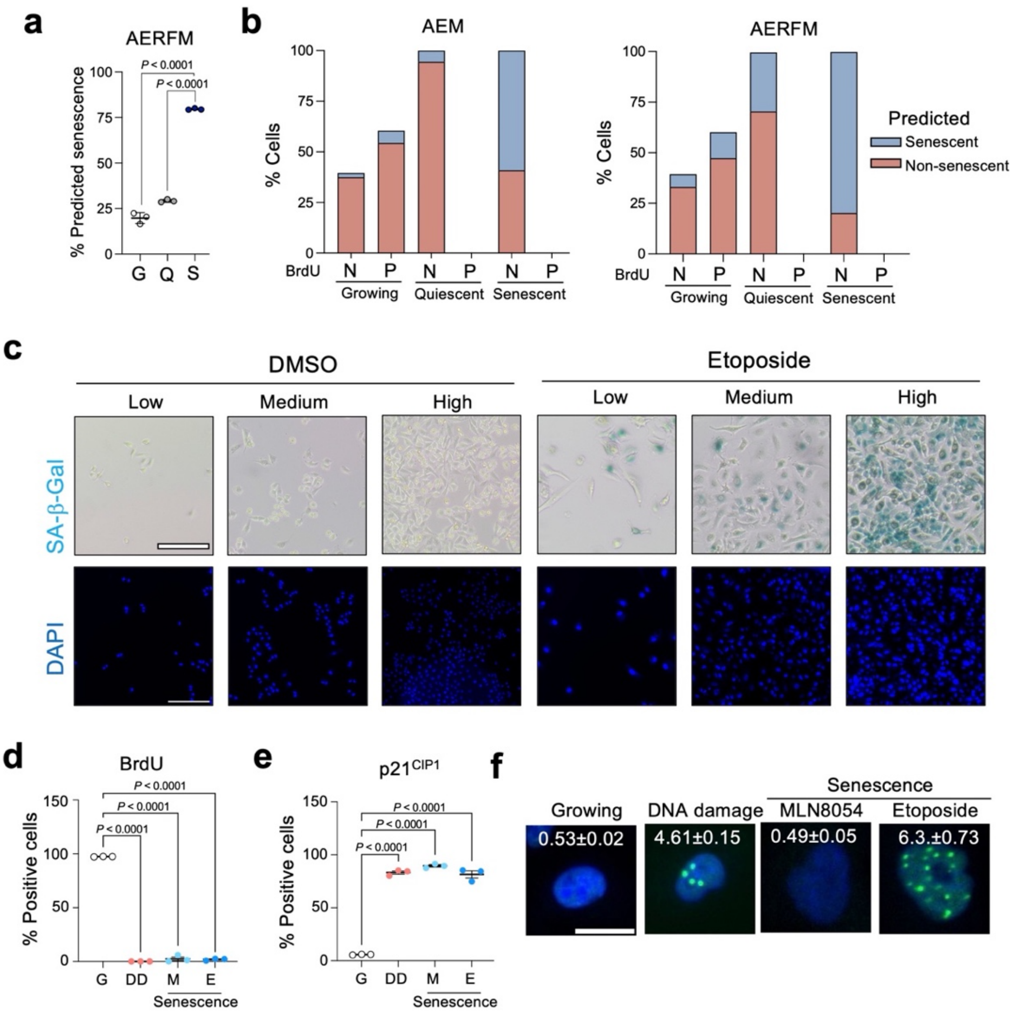

Supplemental Figure 4. Classifiers distinguish senescent from quiescent cells. **a**, Percentage of predicted senescent cells using the AERFM classifier in growing (G), quiescent (Q) or senescent (S) cultures of A549 cells. The statistical significance was calculated using one-way ANOVA (Tukey's multiple comparisons test). Data represent mean ±s.d. (n=3). **b**, Percentage of cells predicted to be senescent according to the BrdU incorporation status (N, negative; P, positive), using the AEM model (left) and AERFM model (right). **c**, Representative images of brightfield SA-β-Gal staining (top) and immunofluorescent DAPI staining (bottom) of DMSO and etoposide-treated A549 cells seeded at different concentrations. Scale bar, 200 µm. **d-e,** BrdU (**d**), p21^{Cip1} (**e**) positive cells in cultures of growing (G), irradiated (DNA damage, DD), and senescent (MLN8054, M; etoposide, E) cells (n=3). The statistical significance was calculated using one-way ANOVA (Tukey's multiple comparisons test). **f,** Representative images of 53BP1-stained cells in different conditions with the average ±s.d. of 53BP1 foci per nucleus per condition. Scale bar, 20 µm. n represents the number of replicates. Source Data are provided in the Source Data File.

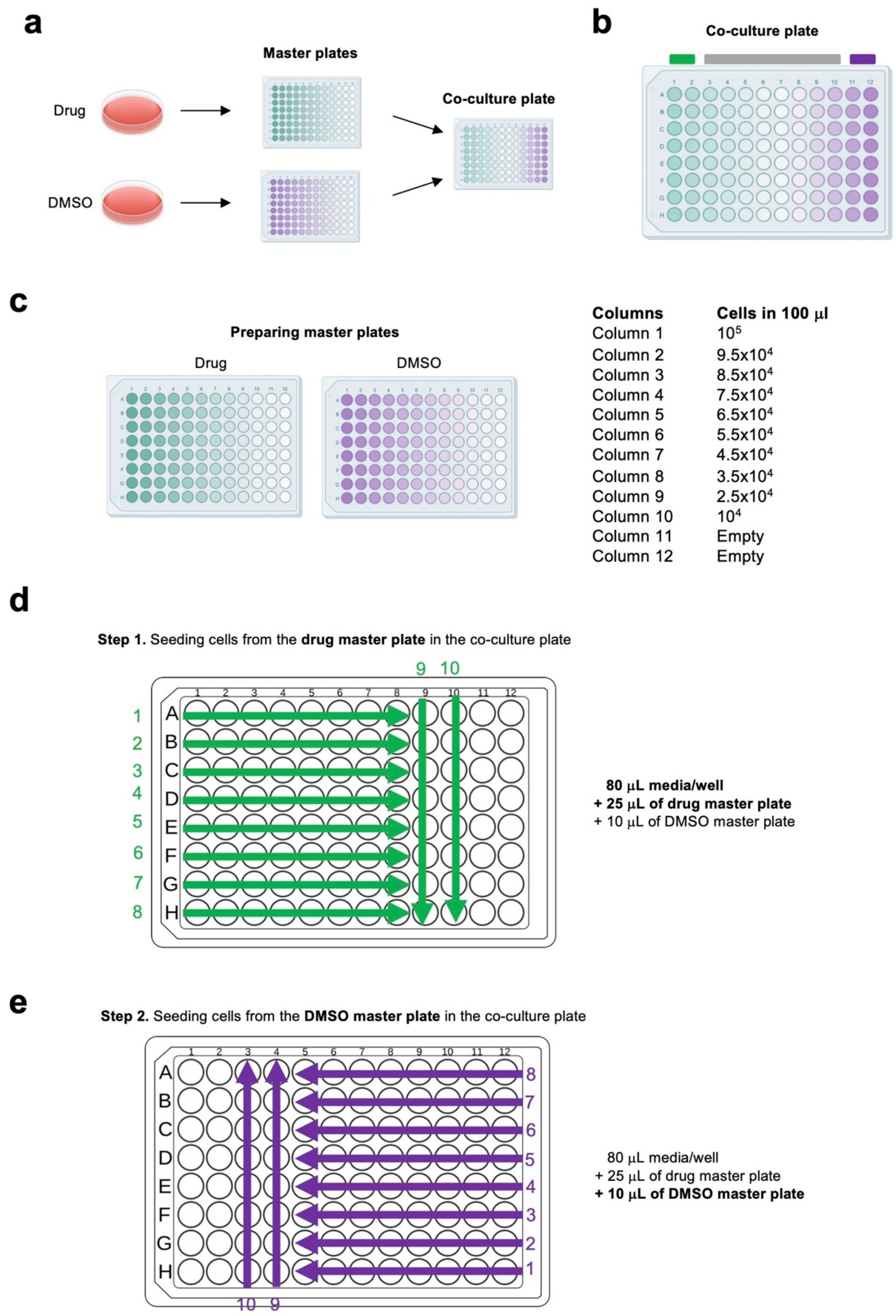

Supplementary Figure 5. Explaining the setup of co-cultures of senescent and non-senescent cells at different ratios. a, Outline. Drug-treated (senescent) or DMSO-treated (normal) cultures were cultured in 100 mm dishes. 6 days after drug treatment, cells were trypsinised, counted, and seeded (as indicated in c) in the drug master plate and DMSO master plate respectively. The master plates were used to

seed the co-culture plate in a two-step process (explained in d and e). Cells in the coculture plate were grown for 24 additional hours before being incubated with C_{12}FDG, fixed, stained with DAPI and imaged. **b**, Co-culture plates contain a total of 96 wells: wells in columns 1 and 2 (marked in green) contain only drug-treated cells; columns 3-10 contain different ratios of drug- and DMSO-treated cells; columns 11 and 12 contain only DMSO-treated cells. Each well is processed as an independent sample in the analysis. Wells with less than 2% of C_{12}FDG (reporting for SA-β-Gal activity)-positive cells are excluded from the analysis. **c**, Preparing the DMSO and drug master plates. 6 days after DMSO or drug treatment, cells were trypsinised, counted and the indicated number of cells were added (in a total volume of 100 μL) to the 8 wells of columns 1-10 (wells in columns 11 and 12 were left empty) of U-bottom 96 well plates. **d-e**, Preparing the co-culture plate. **d**, Step one. 80 μL of growing media were aliquoted per well of a 96-well flat bottom cell culture plate. Then, 25 μL of the drug master plate was added to the indicated wells. Green numbers indicate the column of the drug master plate added to each column or row of the co-culture plate. The arrows indicate the direction in which they were added (taken from row A to row H of the drug master plate). **e**, Step two. 10 μL of the DMSO master plate were added to the indicated wells. Purple numbers indicate the column of the DMSO master plate added to each column or row of the co-culture plate. The arrows indicate the direction in which they were added (taken from row A to row H of the DMSO master plate).

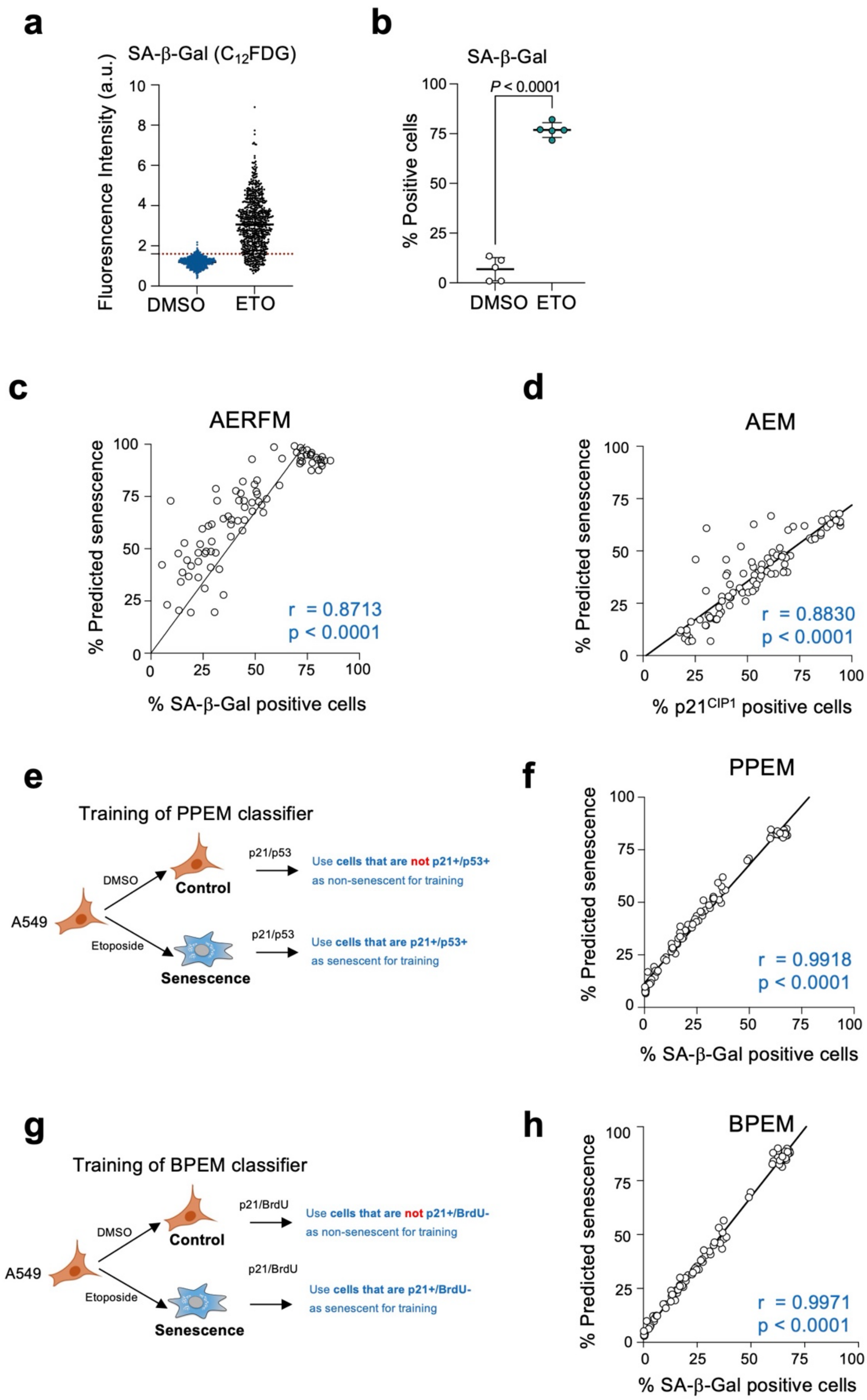

Supplemental Figure 6. Senescence classifiers identify senescence at the single-cell level. a, Levels of SA-β-galactosidase activity (SA-β-Gal) in normal (DMSO) and senescent (etoposide-treated) A549 cells as measured using C_{12}FDG. Red dotted line, arbitrary cut-off to define positive cells. Data from a representative experiment out of 3 (n=750 cells). **b**, Percentage of SA-β-Gal positive cells measured

using C_{12}FDG staining and the threshold shown in a (n=5). Statistical significance was calculated using unpaired, two-tailed, Student's *t*-test. Data represent mean ±s.d. **c-d**, Correlation between the percentage of SA-β-Gal positive cells and percentage of cells predicted to be senescent, using the AERFM classifier (**c**) (r=0.8713; p value<0.0001) and AEM (**d**) (r=0.8830; p <0.0001). **e**, Experimental design for the development of senescence classifiers trained using cells double positive for $p21^{CIP1}$ and p53 (PPEM). Correlation between the percentage of SA-β-Gal positive cells and the percentage of cells predicted to be senescent using the PPEM classifier (r=0.9918; p <0.0001). **g**, Experimental design for the development of senescence classifiers trained using $p21^{CIP1}$ positive/BrdU negative cells (BPEM). **h**, Correlation between percentage of SA-β-Gal positive cells and percentage of cells predicted to be senescent, using the BPEM classifier (r=0.9971; p <0.0001). Correlations in c, d, f, and h were calculated with Pearson correlation coefficients (two-tailed, 95% CI). p-value represents two-tailed nonparametric correlation probability. n=70-96 wells except indicated otherwise. Wells are co-cultures of senescence and non-senescent cells at different ratios as explained in methods. Source Data are provided in the Source Data File.

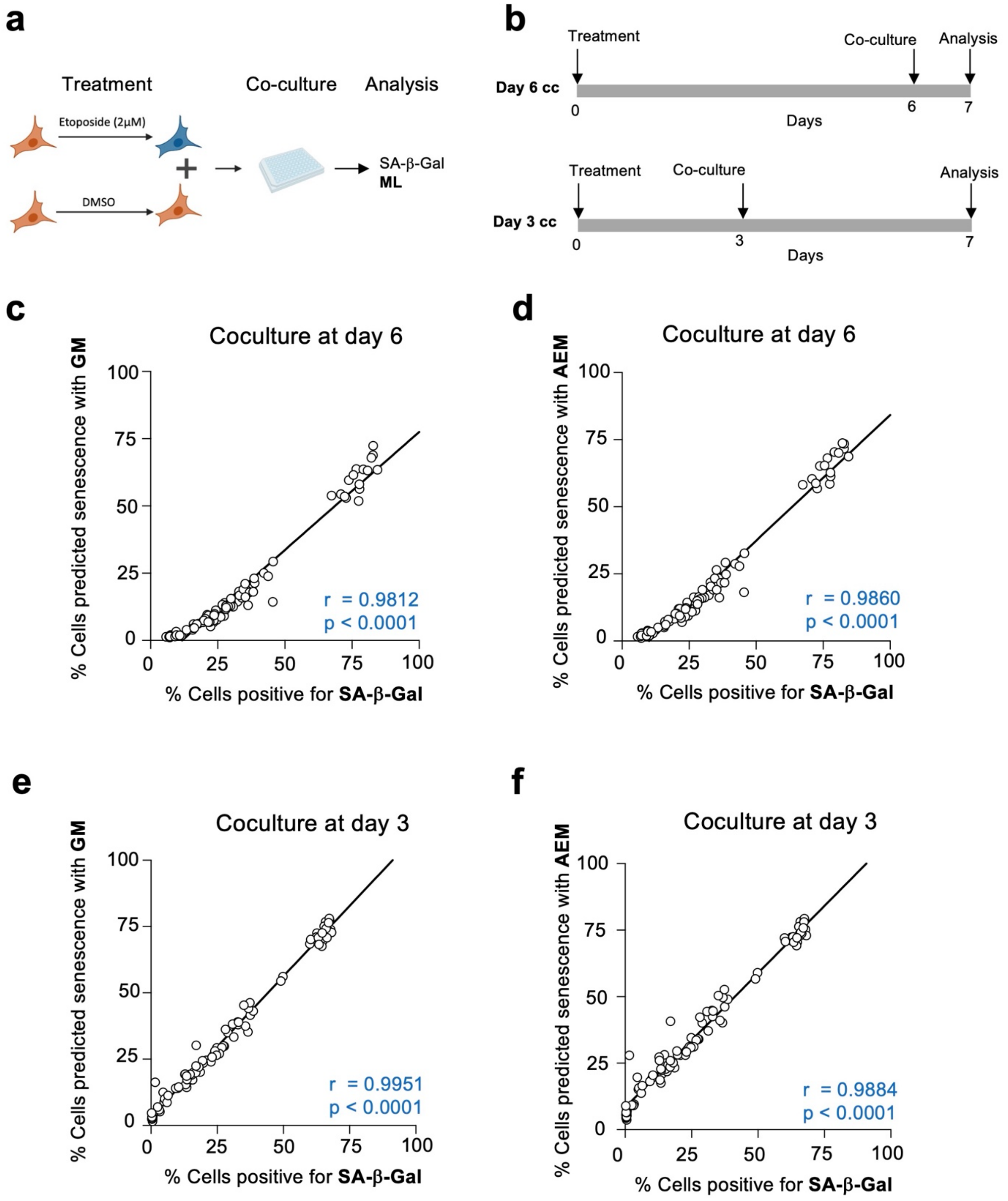

Supplemental Figure 7. Co-culturing senescent and normal cells from day 3 or day 6 does not influence the classifier results. a, Design for the experiments analysing co-cultures of DMSO-treated (normal) or etoposide-treated (senescent) A549 cells and fluorescent SA-β-galactosidase (SA-β-Gal) staining. **b**, Experimental timepoint design for experiments where cells were co-cultured (cc) at day 6 (day 6 cc), as in the rest of experiments in this manuscript or at day 3 (day 3 cc). **c-d**, Correlation between SA-β-Gal positive cells and cells predicted to be senescent in etoposide-

treated A549 cells, using GM (**c**) (r=0.9812; p <0.0001) and AEM (**d**) (r=0.9860; p < 0.0001) classifiers after co-seeding at day 6. **e-f**, Correlation between SA-β-Gal positive cells and cells predicted to be senescent in etoposide-treated A549 cells, using GM (**e**) (r=0.9951; p <0.0001) and AEM (**f**) (r=0.9884; p <0.0001) classifiers after co-seeding at day 3. Pearson correlation coefficient (two-tailed, 95% CI). p-value represents two-tailed nonparametric correlation probability. n=70-96 wells. Wells are co-cultures of senescent and non-senescent cells at different ratios as explained in methods. Source Data are provided in the Source Data File.

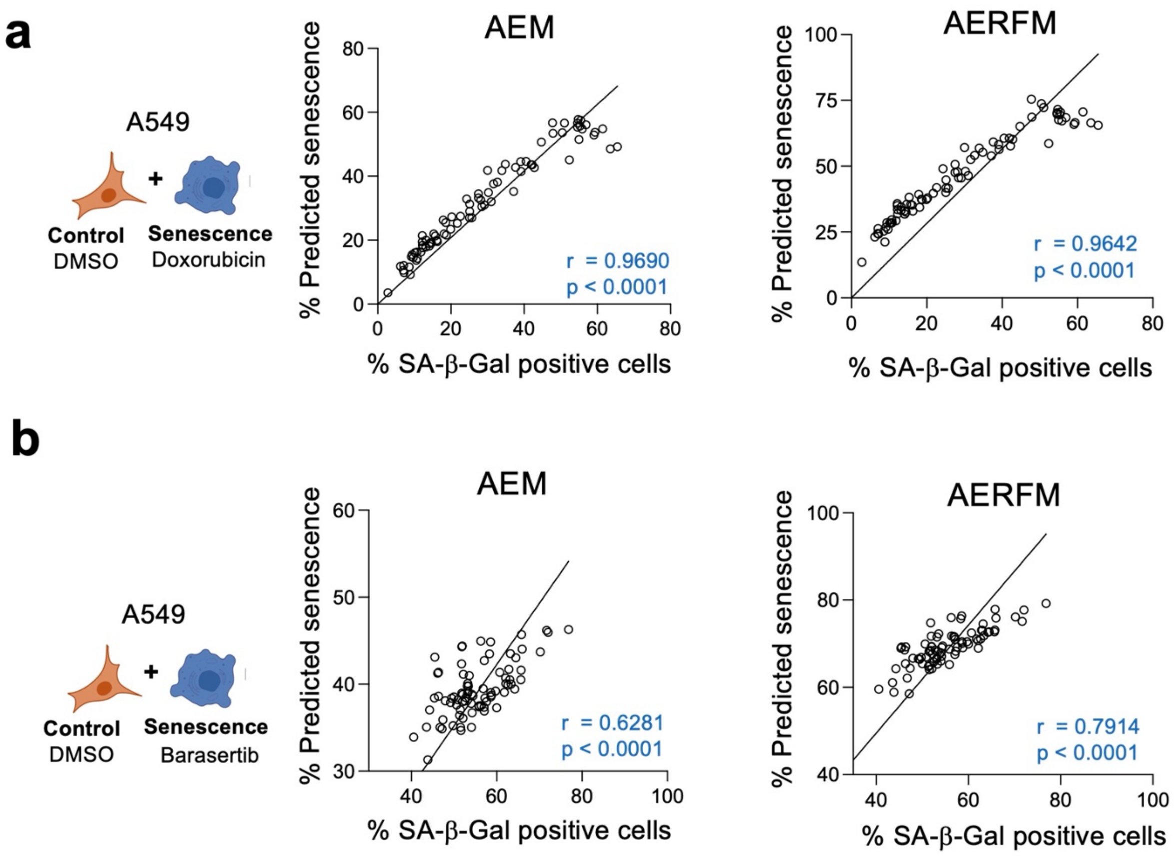

Supplemental Figure 8. Correlation between predicted and senescent cells in co-cultures of senescent and non-senescent A549 cells. a Correlation between SA-β-galactosidase (SA-β-Gal) positive cells and cells predicted to be senescent in doxorubicin-treated A549 cells, using AEM (r=0.9690; p<0.0001) and AERFM (r=0.9642; p< 0.0001) classifiers. **b**, Correlation between SA-β-Gal positive cells and cells predicted to be senescent in barasertib-treated A549 cells, using AEM (r=0.6281; p<0.0001) and AERFM (r=0.7914; p<0.0001) classifiers. Pearson correlation coefficient (two-tailed, 95% CI) was used. p-value represents two-tailed nonparametric correlation probability. n=70-96 wells. Wells are co-cultures of senescence and non-senescent cells at different ratios as explained in methods. Source Data are provided in the Source Data File.

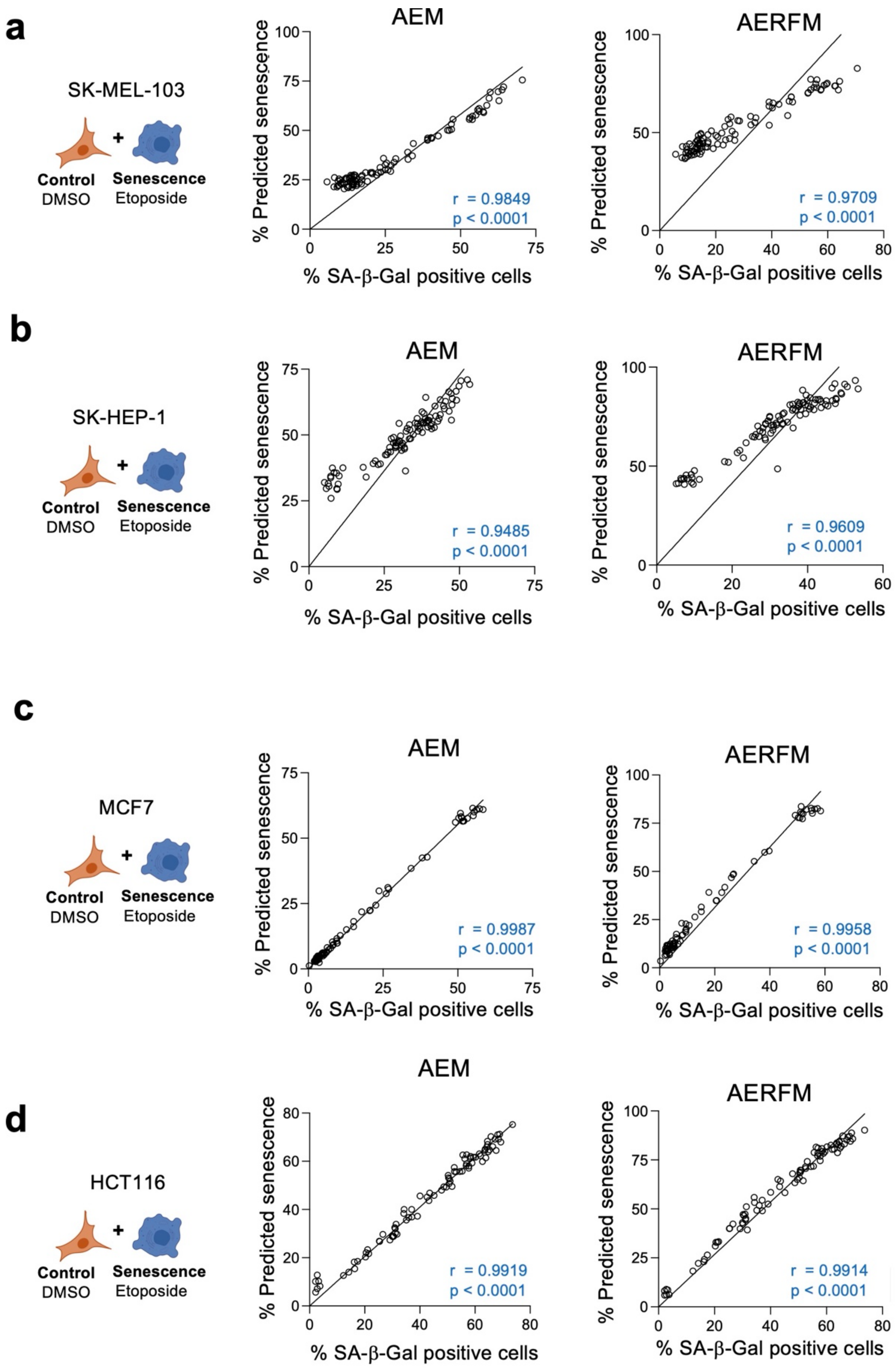

Supplemental Figure 9. Correlation between predicted and senescent cells in co-cultures of different senescent and non-senescent cancer cells. a, Correlation between SA-β-galactosidase (SA-β-Gal) positive cells and cells predicted to be senescent in etoposide-treated SK-MEL-103 cells, using AEM ($r=0.9849$; $p<0.0001$) and AERFM ($r=0.9709$; $p<0.0001$) classifiers. **b**, Correlation between SA-β-Gal

positive cells and cells predicted to be senescent in etoposide-treated SK-HEP-1 cells, using AEM (r=0.9485; p<0.0001) and AERFM (r=0.9609; p<0.0001) classifiers. **c**, Correlation between SA-β-Gal positive cells and cells predicted to be senescent in etoposide-treated MCF7 cells, using AEM (r=0.9987; p<0.0001) and AERFM (r=0.9958; p<0.0001) classifiers. **d**, Correlation between SA-β-Gal positive cells and cells predicted to be senescent in etoposide-treated HCT116 cells, using AEM (r=0.9919; p<0.0001) and AERFM (r=0.9914; p<0.0001) classifiers. Pearson correlation coefficient (two-tailed, 95% CI) was used. p-value represents two-tailed nonparametric correlation probability. n=70-96 wells. Wells are co-cultures of senescence and non-senescent cells at different ratios as explained in methods. Source Data are provided in the Source Data File.

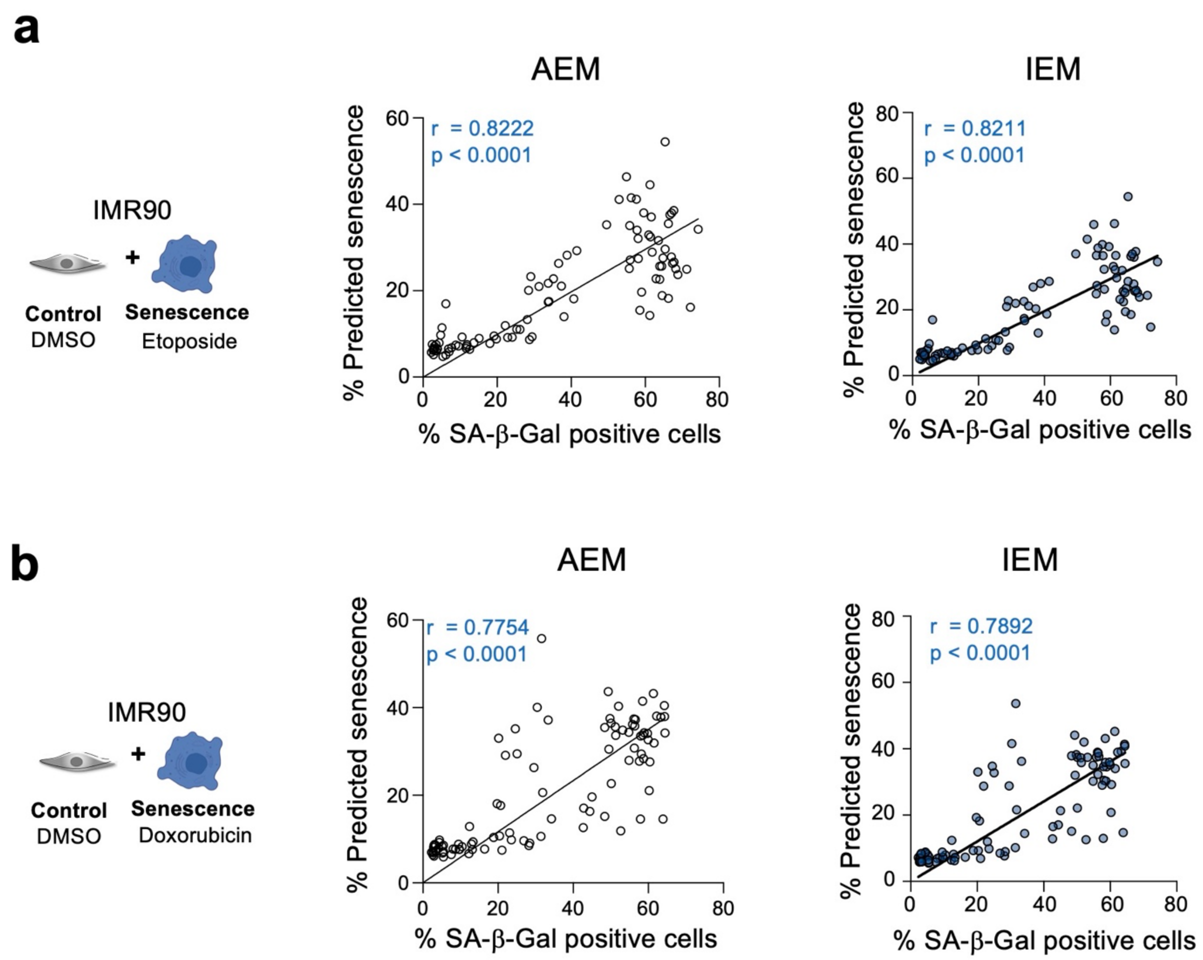

Supplemental Figure 10. Correlation between predicted and senescent cells in co-cultures of senescent and non-senescent IMR90 cells. a, Schematic of senescence detection experiment (left); Correlation between SA-β-galactosidase (SA-β-Gal) positive cells (x-axis) and cells predicted to be senescent (y-axis) in etoposide-treated IMR90 cells, using AEM ($r=0.8222$; $p<0.0001$) and IEM ($r=0.8211$; $p<0.0001$) classifiers. **b**, Correlation between SA-β-Gal positive cells and cells predicted to be senescent in doxorubicin-treated IMR90 cells, using AEM ($r=0.7754$; $p<0.0001$) and IEM ($r=0.7892$; $p<0.0001$) classifiers. AEM, A549 etoposide model; IEM, IMR90 etoposide model. Pearson correlation coefficient (two-tailed, 95% CI) was used. p-value represents two-tailed nonparametric correlation probability. n=70-96 wells. Wells are co-cultures of senescence and non-senescent cells at different ratios as explained in methods. Source Data are provided in the Source Data File.

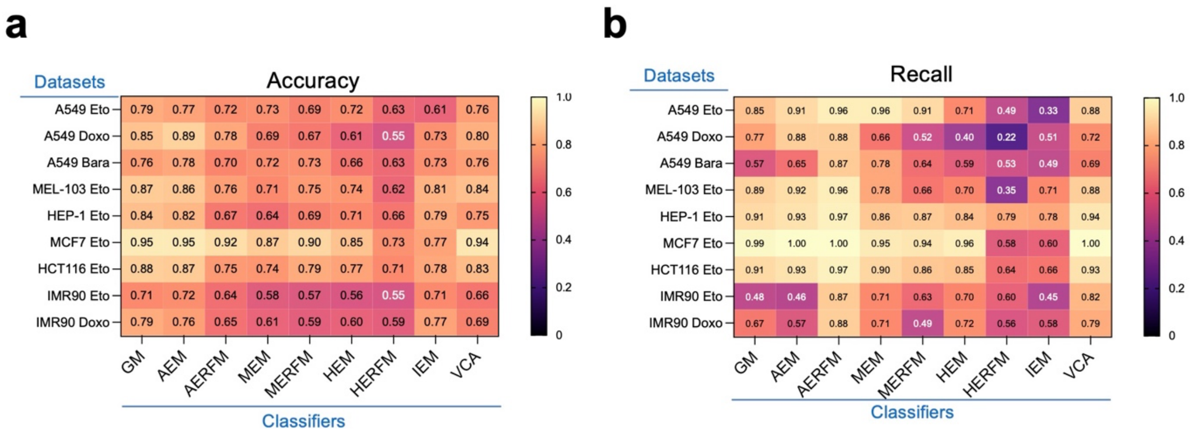

Supplemental Figure 11. Comparison of the performance of different senescence classifiers. a-b, Accuracy (**a**), and recall (**b**) of senescence classifiers (x-axis) in the different datasets (derived from co-cultures of different types of senescent cells with non-senescent counterparts at different ratios as explained in methods) represented in heatmaps. Each heatmap contains 9 classifiers: 8 algorithms (GM, AEM, AERFM, MEM, MERFM, HEM, HERFM, and IEM) and the last column corresponds to a voting-based consensus algorithm (VCA), obtained by equal weight voting system of the previous 8 algorithms. Accuracy and recall measures represent experiment median values, calculated from n=70-96 wells each. Source Data are provided in the Source Data File.

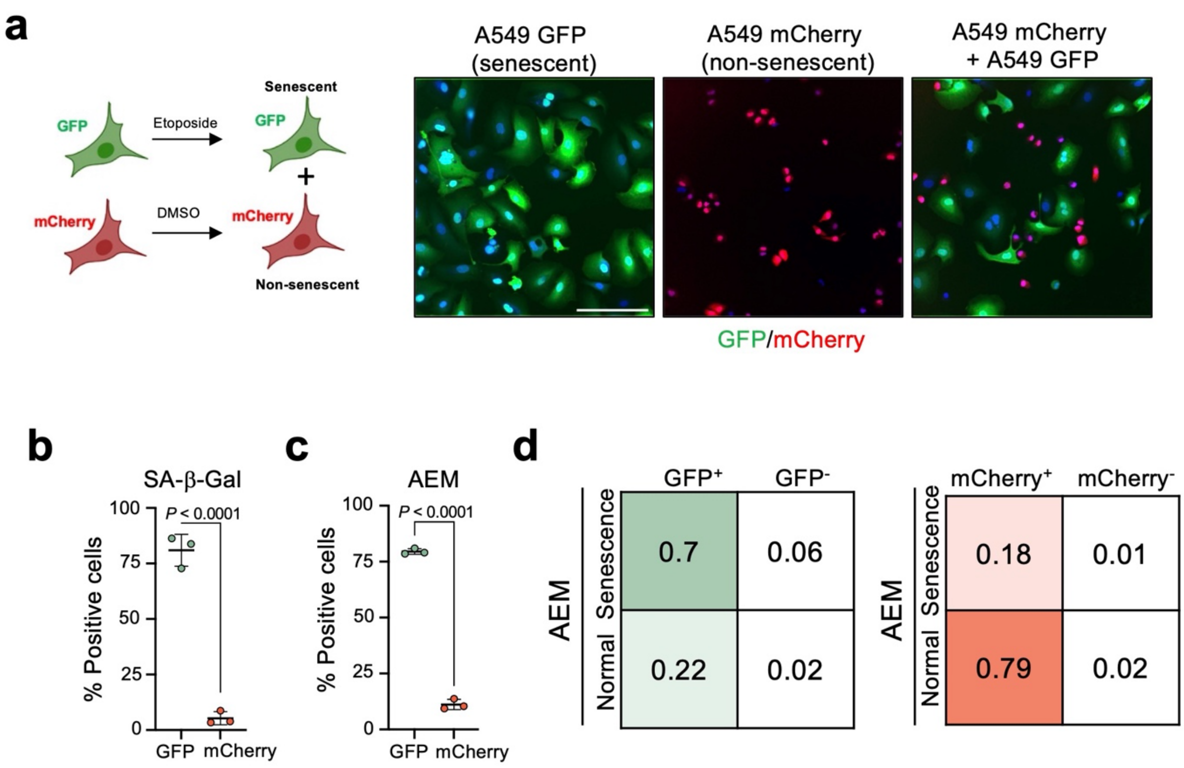

Supplemental Figure 12. Using cocultures of etoposide-treated A549 GFP and DMSO-treated A549 mCherry cells to set up senolytic assays. a, Schematic of the set-up for the senolytic experiments. Representative immunofluorescence images of A549 GFP, A549 mCherry cells and a coculture. Scale bar, 200 µm. **b,** SA-β-galactosidase (SA-β-Gal) staining in etoposide-treated A549 (GFP) and DMSO-treated A549 (mCherry) cells (n=3). **c,** Quantification of cells predicted to be senescent in etoposide-treated A549 (GFP) and DMSO-treated A549 (mCherry) cells (n=3). d, Confusion matrices for GFP (left) and mCherry controls (right), with triplicate averages plotted per condition. Statistical significance was calculated using unpaired, two-tailed, Student's *t*-tests. Data represent mean ± s.d; n represents the number of replicates. Source Data are provided in the Source Data File.

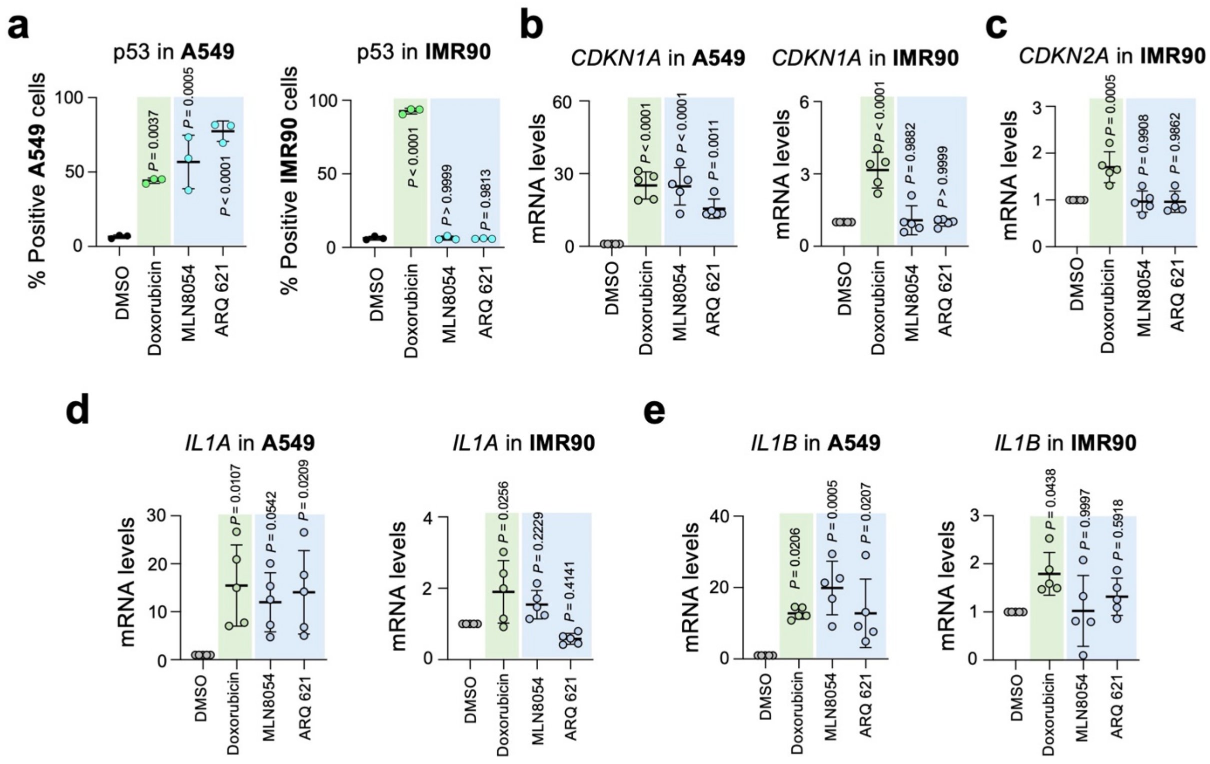

Supplemental Figure 13. Induction of senescence by drugs identified on the screen. a, Quantification of p53 expression as assessed by immunofluorescence in A549 (left) and IMR90 (right) after treatment with DMSO, doxorubicin, MLN8054 and ARQ621 (n=3). **b,** mRNA expression levels of *CDKN1A* in A549 and IMR90 cells respectively (n=5). **c,** mRNA expression levels of *CDKN2A* in IMR90 cells (n=5). **d-e**, *IL1A* (**d**) and *IL1B* (**e**) mRNA expression levels in A549 and IMR90 cells (n=5). Significance was calculated with a one-way ANOVA (Tukey's multiple comparisons test). Data represent mean ±s.d. n represents the number of replicates. Source Data are provided in the Source Data File.

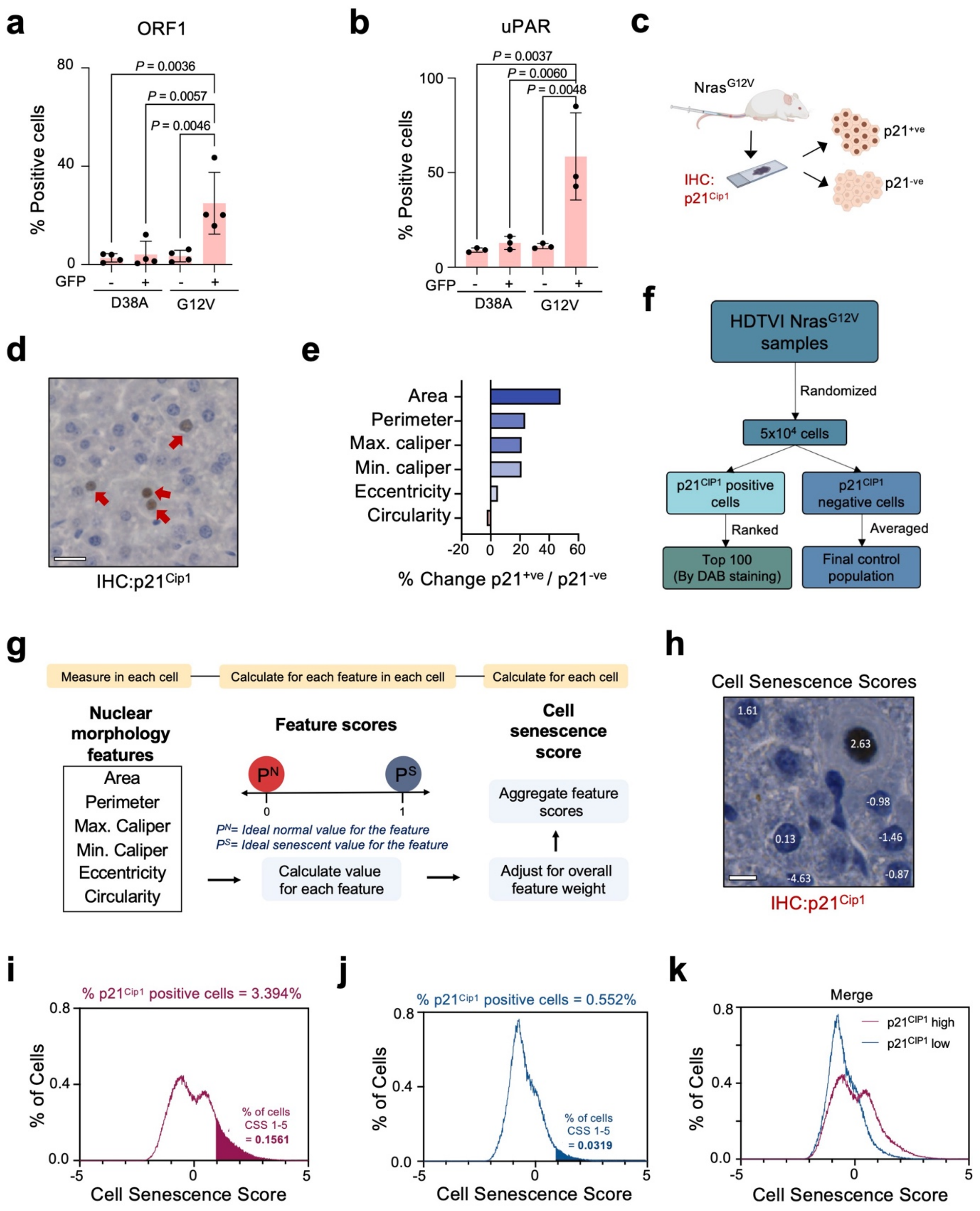

Supplemental Figure 14. Calculating cell and tissue senescence scores from tissue sections. a-b, Quantification of cells positive for ORF1 (**a**, n=4 per group) and uPAR (**b**, n=3 per group) in liver sections derived from mice transduced with $Nras^{G12V}$-ires-GFP or $Nras^{G12V, D38A}$-ires-GFP transposons as analysed by immunofluorescence. GFP is co-expressed with Nras mutants. Significance was calculated with a one-way ANOVA (Tukey's multiple comparisons test). Data represent mean ±s.d. n=mice. **c**,

Schematic for p21^{Cip1}-positive and negative population feature extraction of liver sections of mice transducing with Nras^{G12V}-expressing vectors. **d**, Representative p21^{Cip1}-stained liver sample. Red arrows indicate p21^{Cip1}-positive, senescent hepatocytes. Scale bar, 20 μm. **e**, Difference in the nuclear features of the p21^{Cip1}-positive and the p21^{Cip1}-negative population, as percentual change. **f**, Schematic for the development of the normal and senescent (p21^{Cip1}-positive) training datasets. **g**, Schematic for assigning the cell senescence scores (CSS). **h**, Representative p21^{Cip1}-stained tissue section with individual cell senescence score (CSS) indicated in each cell. CSS values of 1 or more indicate senescence. Scale bar, 10 μm. **i-j**, Single-cell senescence score distribution in samples with relatively high p21^{Cip1} (p21^{Cip1}-positive cells= 3.394%) (**i**) and relatively low p21^{Cip1} (p21^{Cip1}-positive cells= 0.552%) (**j**) samples. The percentage of cells between 1 to 5 is indicated and was used to define the tissue senescence score. **k**, Merged single-cell senescence score distribution for the tissues shown in **i** and **j**. Source Data are provided in the Source Data File.

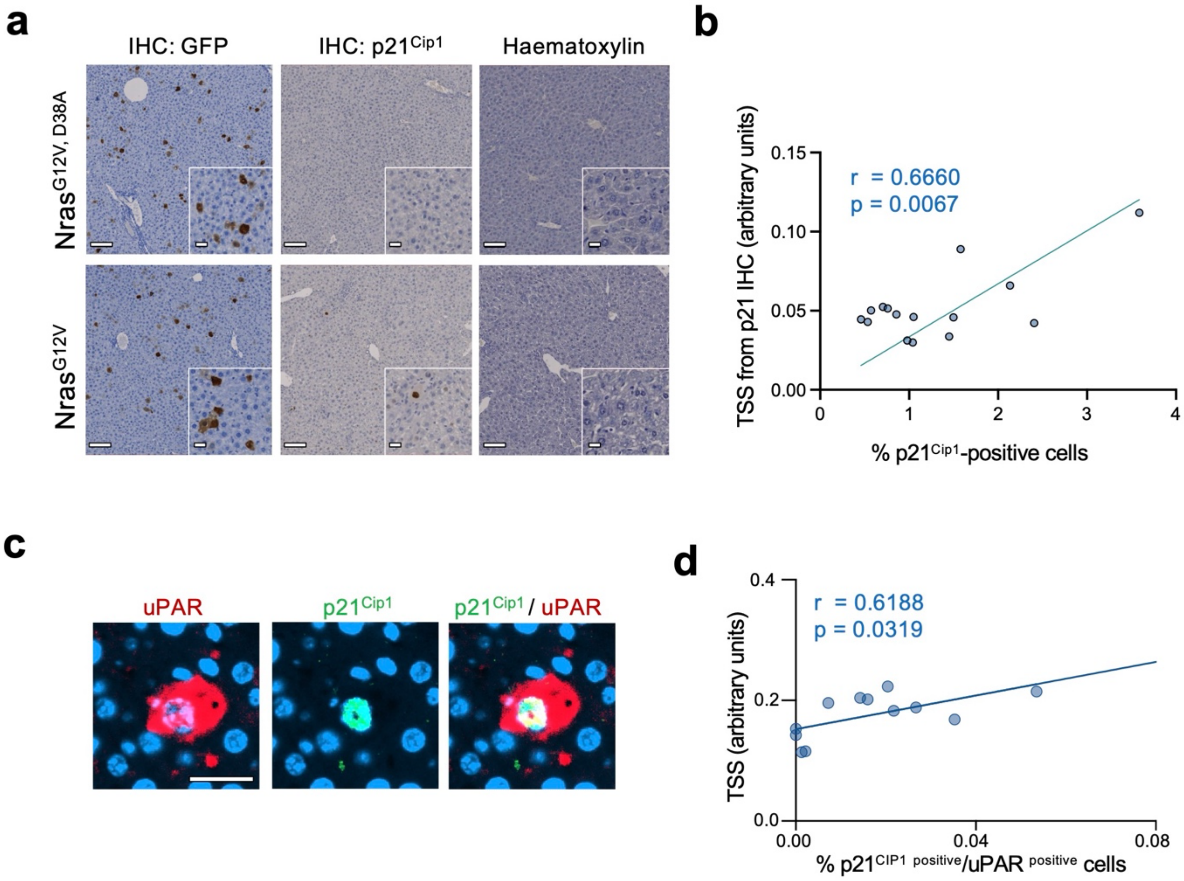

Supplemental Figure 15. A model of oncogene-induced senescence during liver cancer initiation. **a**, Representative images of GFP, p21^{Cip1} and haematoxylin staining in liver sections in liver sections derived from mice transduced with Nras^{G12V}-ires-GFP or Nras$^{G12V,\ D38A}$-ires-GFP transposons. Scale bars are 100µm in the main picture and 20 µm for the zoomed section. **b**, Pearson correlation coefficient (two-tailed, 95% CI) between tissue senescence score calculated in haematoxylin-stained and p21^{Cip1}-stained liver sections (n=15). p-value represents two-tailed nonparametric correlation probability. **c**, Representative images of immunofluorescent p21^{Cip1}/uPAR co-staining in liver sections of mice transduced with a transposon expressing Nras^{G12V}. Scale bar, 50µm. **d**, Pearson correlation coefficient (two-tailed, 95% CI) between tissue senescence score calculated in haematoxylin-stained and p21^{Cip1}/uPAR stained liver sections (n=12). P-value represents two-tailed nonparametric correlation probability. n represents the number of mice. Source Data are provided in the Source Data File.

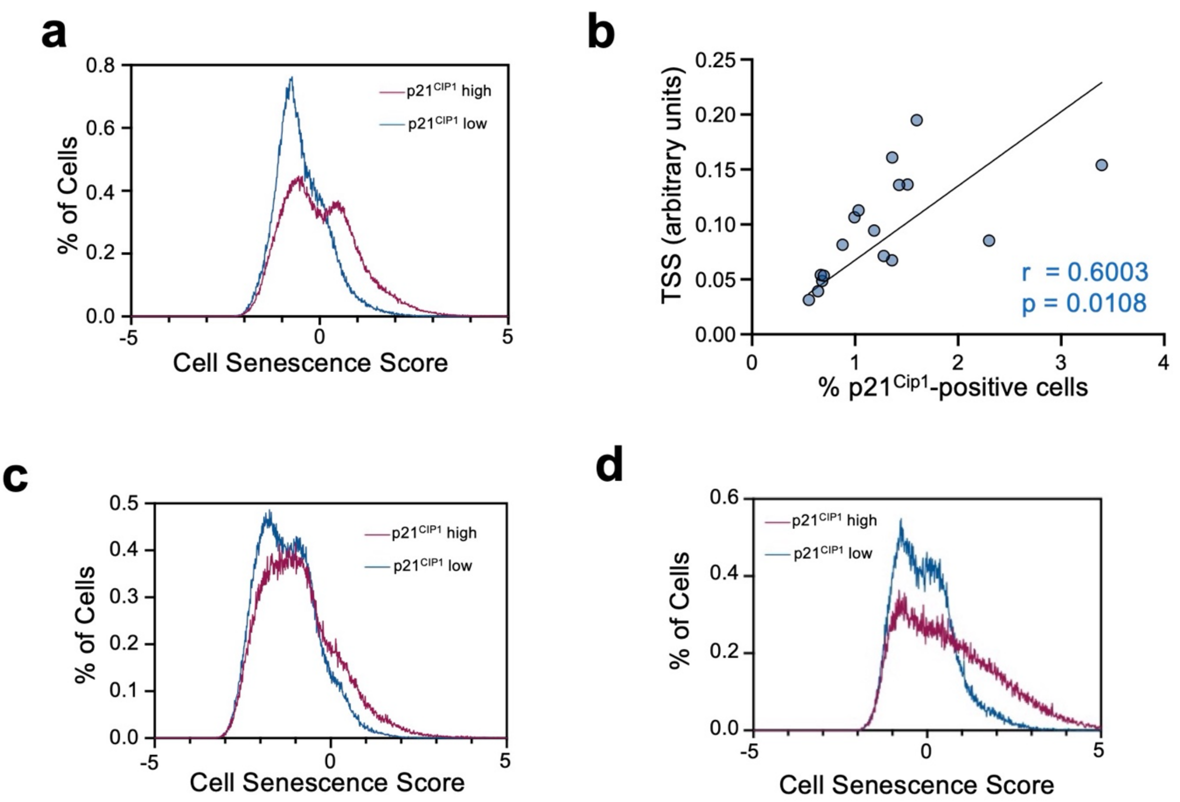

Supplementary Figure 16. Calculating cell and tissue senescence scores in models of senolysis, liver fibrosis and ageing. a, Distribution of cell senescence score in two sections corresponding to p21^{Cip1} high (red) and low (blue) samples of the senolysis experiment. **b**, Pearson correlation coefficient (two-tailed, 95% CI) between the TSS and percentage of p21^{Cip1} positive cells for samples of the senolysis experiment (n=17 mice). P-value represents two-tailed nonparametric correlation probability. **c**, Distribution of cell senescence score in two sections corresponding to p21^{Cip1} high (red) and low (blue) samples of the liver fibrosis experiment. **d**, Distribution of cell senescence score in two sections corresponding to p21^{Cip1} high (red) and low (blue) samples of the ageing experiment. Source Data are provided in the Source Data File.

SUPPLEMENTARY TABLES

Supplementary Table 1. Datasets used to train the senescence classifiers.

Cell type	Senescence inducer	Library characteristics	Selected senescent cells	Name
**A549**	Etoposide	$2.23x10^5$ DMSO cells<br>$1.01x10^6$ TIS cells	All	A549 Eto
**A549**	Etoposide	$0.34x10^5$ DMSO cells<br>$1.45x10^5$ TIS cells	All	A549 Eto CP
**A549**	Etoposide	$2.77x10^5$ DMSO cells<br>$2.92x10^5$ TIS cells	SA-β-Gal +	A549 Eto SA-β-Gal
**A549**	Etoposide	$6.65x10^4$ DMSO cells<br>$2.87x10^5$ TIS cells	BrdU- / p21 +	A549 Eto Brdu/p21
**A549**	Etoposide	$2.60x10^4$ DMSO cells<br>$2.63x10^5$ TIS cells	p21+ / p53+	A549 Eto p21-p53
**SK-MEL-103**	Etoposide	$6.66x10^5$ DMSO cells<br>$4.76x10^5$ TIS cells	All	SK-MEL-103 Eto
**SK-HEP-1**	Etoposide	$2.07x10^5$ DMSO cells<br>$5.05x10^5$ TIS cells	All	SK-HEP-1 Eto
**IMR90**	Etoposide	$9.88x10^5$ DMSO cells<br>$8.2x10^5$ TIS cells	All	IMR90 Eto
**A549 + SK-HEP-1 + SK-MEL-103**	Eto + Doxo + Ali + Bara	10^4 randomized cells /condition (Etoposide, Doxorubicin, Alisertib, Barasertib) and per cell line (A549, SK-MEL-103, SK-HEP-1), for a total of $1.2x10^5$ treated and $1.2x10^5$ DMSO-containing training set	All	General

Supplementary Table 2. Senescence classifiers used in this study.

Name	Training set	Algorithm
**AEM**	A549 Eto	Classification tree
**AECP**	A549 Eto CP	Classification tree
**BAEM**	A549 Eto SA-β-Gal	Classification tree
**BPEM**	A549 Eto Brdu/p21	Classification tree
**PPEM**	A549 Eto p21/p53	Classification tree
**MEM**	SK-MEL-103 Eto	Classification tree
**HEM**	SK-HEP-1 Eto	Classification tree
**IEM**	IMR90 Eto	Classification tree
**GM**	General	Classification tree
**AERFM**	A549 Eto	Random forest
**AERFCP**	A549 Eto CP	Random forest
**MERFM**	SK-MEL-103 Eto	Random forest
**HERFM**	SK-HEP-1 Eto	Random forest
**Voting-based clustering algorithm (VCA)**	n/a	Voting-based clustering algorithm

Supplementary Table 3. SA-β-galactosidase activity in the models of senescence used in this study.

Cell type	Senescence inducer	% SA-β-Gal positive cells (Average ± SD; n=3)
**A549**	Etoposide	93.72 ± 5.12
**A549**	Doxorubicin	90.23 ± 5.31
**A549**	Barasertib	88.82 ± 2.01
**SK-MEL-103**	Etoposide	92.36 ± 2.79
**SK-HEP-1**	Etoposide	91.72 ± 1.97
**MCF7**	Etoposide	74.24 ± 1.52
**HCT116**	Etoposide	86.76 ± 3.78
**IMR90**	Etoposide	86.20 ± 5.16
**IMR90**	Doxorubicin	85.56 ± 5.32

Supplementary Table 4. Effect of changes in the CSS range on the correlation between TSS and percentage of senescent cells.

	Liver cancer initiation[1]		Senolysis[2]		Fibrosis[3]	
**CSS range**	**Correlation**	**P- value**	**Correlation**	**P- value**	**Correlation**	**P- value**
1 to 5	0.67	0.0067	0.5	0.0419	0.52	0.0684
1 to 4	0.66	0.008	0.5	0.0416	0.52	0.0684
1 to 3	0.66	0.0124	0.5	0.0424	0.52	0.0673
1 to 2	0.56	0.0287	0.5	0.0418	0.54	0.0589
2 to 3	0.74	0.0018	0.48	0.049	0.47	0.103
2 to 4	0.76	0.001	0.49	0.0473	0.47	0.1026
4 to 5	0.8	0.0003	0.35	0.1638	0.46	0.1095
0 - 1	0.19	0.5028	0.5	0.0394	0.39	0.1847
0 - 2	0.37	0.1729	0.5	0.0378	0.48	0.0962
0 -3	0.45	0.0935	0.5	0.0371	0.49	0.0869
0 - 4	0.48	0.0695	0.51	0.0364	0.5	0.0799
0 - 5	0.49	0.0616	0.51	0.0365	0.51	0.0775

[1]Liver cancer initiation experiment (Figure 7e-k and Sup Figure 15)
[2]Senolysis experiment (Figure 8a-d)
[3]Fibrosis experiment (Figure 8e-h)
Pearson correlation coefficient (two-tailed, 95% CI) was performed for all conditions. Source Data are provided in the Source Data File.

Supplementary Table 5. Parameter values for features used in the senescence scoring system used in tissue sections.

Feature	P^N	P^S
Area	38.8972	57.4797
Perimeter	24.3416	30.0912
Circularity	0.7961	0.7746
Max caliper	8.6617	10.4962
Min caliper	6.0919	7.3743
Eccentricity	0.6511	0.6833
