# Supplementary material for: Detection of senescence using machine learning algorithms based on nuclear features
Source: Nat Commun. 2024 Feb 3;15:1041. doi: 10.1038/s41467-024-45421-w (PMC10838307; doi:10.1038/s41467-024-45421-w)
Supplement: Supplementary file 3 — Reporting Summary [file 41467_2024_45421_MOESM3_ESM.pdf]

nature portfolio

Corresponding author(s): Jesus Gil

Last updated by author(s): Jan 12, 2024

Reporting Summary

Nature Portfolio wishes to improve the reproducibility of the work that we publish. This form provides structure for consistency and transparency in reporting. For further information on Nature Portfolio policies, see our Editorial Policies and the Editorial Policy Checklist.

Statistics

For all statistical analyses, confirm that the following items are present in the figure legend, table legend, main text, or Methods section.

n/a	Confirmed	
☐	☒	The exact sample size (*n*) for each experimental group/condition, given as a discrete number and unit of measurement
☐	☒	A statement on whether measurements were taken from distinct samples or whether the same sample was measured repeatedly
☐	☒	The statistical test(s) used AND whether they are one- or two-sided<br>*Only common tests should be described solely by name; describe more complex techniques in the Methods section.*
☒	☐	A description of all covariates tested
☐	☒	A description of any assumptions or corrections, such as tests of normality and adjustment for multiple comparisons
☐	☒	A full description of the statistical parameters including central tendency (e.g. means) or other basic estimates (e.g. regression coefficient) AND variation (e.g. standard deviation) or associated estimates of uncertainty (e.g. confidence intervals)
☐	☒	For null hypothesis testing, the test statistic (e.g. *F*, *t*, *r*) with confidence intervals, effect sizes, degrees of freedom and *P* value noted<br>*Give P values as exact values whenever suitable.*
☒	☐	For Bayesian analysis, information on the choice of priors and Markov chain Monte Carlo settings
☐	☒	For hierarchical and complex designs, identification of the appropriate level for tests and full reporting of outcomes
☒	☐	Estimates of effect sizes (e.g. Cohen's *d*, Pearson's *r*), indicating how they were calculated

Our web collection on statistics for biologists contains articles on many of the points above.

Software and code

Policy information about availability of computer code

Data collection	In Cell Analyzer 2500 HS (Cytiva)<br>Zeiss AxioScan Z.1 or Leica Aperio AT2 slide scanner<br>CFX96 RT-PCR system C1000 Touch (Bio-Rad)
Data analysis	GraphPad Prism 9 version 9.4.0 (453) for macOS Catalina (version 10.15.7) was used for statistical analysis<br>QuPath version (0.3.0)<br>CellProfiler 4.2.1<br>In Carta (Cytiva, version 1.14)<br>ZZeiss LSM software Zen 2 Blue<br>Sublime Text (Version Build 4126)<br>Python version 3.7.7<br>B score calculation:<br>R Studio (Version 2023.06.2+561) R (version 4.3.1) and packages BiocManager (version 1.30.22) and cellHTS2 (version 2.64) were used.<br>(https://bioconductor.org/packages/release/bioc/html/cellHTS2.html)<br>(10.1186/1471-2105-11-185)<br><br>Custom code and training sets used in this project can be found at:<br>https://github.com/Sen-Lab-LMS/Senescence_nuclear_features, which is archived in Zenodo with the identifier [https://zenodo.org/doi/10.5281/zenodo.10499895].<br><br>Python modules utilized:

sklearn.model_selection/train_test_split; sklearn.ensemble/RandomForestClassifier; sklearn/metrics/roc_curve, roc_auc_curve, precision_score, confusion_matrix, plot_precision_recall_curve, PrecisionRecallDisplay; numpy/mean; numpy/std; pandas; matplotlib.pyplot; seaborn; graphviz; export_text; import tree; DecisionTreeClassifier; plot_tree; train_test_split; cross_val_score; confusion_matrix; plot_confusion_matrix; metrics; roc_curve; RocCurveDisplay; scikitplot/skplt

For manuscripts utilizing custom algorithms or software that are central to the research but not yet described in published literature, software must be made available to editors and reviewers. We strongly encourage code deposition in a community repository (e.g. GitHub). See the Nature Portfolio guidelines for submitting code & software for further information.

Data

Policy information about availability of data

All manuscripts must include a data availability statement. This statement should provide the following information, where applicable:

- Accession codes, unique identifiers, or web links for publicly available datasets
- A description of any restrictions on data availability
- For clinical datasets or third party data, please ensure that the statement adheres to our policy

DATA AVAILABILITY
Source data are provided with this paper. All other data supporting the findings of this study are available from the corresponding author upon reasonable request.

CODE AVAILABILITY
Custom code and training sets can be found at:
https://github.com/Sen-Lab-LMS/Senescence_nuclear_features, which is archived in Zenodo with the identifier [https://zenodo.org/doi/10.5281/zenodo.10499895].

Research involving human participants, their data, or biological material

Policy information about studies with human participants or human data. See also policy information about sex, gender (identity/presentation), and sexual orientation and race, ethnicity and racism.

Reporting on sex and gender	Human liver biopsies were fully anonymised and acquired from the Imperial Hepatology and Gastroenterology Biobank, therefore no regard on sex and gender was considered.
Reporting on race, ethnicity, or other socially relevant groupings	Human liver biopsies were fully anonymised and acquired from the Imperial Hepatology and Gastroenterology Biobank, therefore no regard race, ethnicity of other social relevant groupings was considered.
Population characteristics	They are liver biopsies from patients with non-alcoholic fatty liver disease and are fully anonymised.
Recruitment	Human liver tissue samples part of the Imperial Hepatology and Gastroenterology Biobank. Informed written consent was provided by the donors.
Ethics oversight	REC approved by the Oxford C Research Ethics Committee under REC reference 16/SC/0021. Informed written consent was provided by the donors.

Note that full information on the approval of the study protocol must also be provided in the manuscript.

Field-specific reporting

Please select the one below that is the best fit for your research. If you are not sure, read the appropriate sections before making your selection.

☒ Life sciences ☐ Behavioural & social sciences ☐ Ecological, evolutionary & environmental sciences

For a reference copy of the document with all sections, see nature.com/documents/nr-reporting-summary-flat.pdf

Life sciences study design

All studies must disclose on these points even when the disclosure is negative.

Sample size	No statistical methods were used to pre-determine sample sizes, but our sample sizes are similar to those reported previously (e.g McHugh et al Nat Cell Biol 2023; Guerrero et al Nat Ageing 2022; Guerrero et al Nat Metab 2019; Triana-Martinez et al Nat Comm 2019)
Data exclusions	On the cell co-culture experiments, wells with less than 2% of C12FDG (reporting for SA-b-Gal activity)-positive cells were excluded from the analysis. No mice were excluded in fibrosis, senolysis and ageing experiments. For preneoplastic senescence experiment mice where HDTVI was not successfully performed were excluded from the study (3 mice were excluded). For preneoplastic senescence experiment stainings, 2 mice were excluded for uPAR staining, due to lack of tissue/re-embedding issues that rendered the tissues unusable.

Replication	All experiments were reproducible. Every figure states how many times each experiment was performed with similar results.
Randomization	For the experiment of preneoplastic senescence assessment mice were tagged and randomized in cages separating them in groups before they were weighted or any procedure performed on them. For senolysis experiments, 4 days after HDTVI, mice were bled to assess presence of gaussia luciferase signal in the blood serum and used to randomize groupings for vehicle and drug treated groups. For the fibrosis experiment, mice were weighed at Day 0 and divided into the two groups based on their weight so that both the Oil and Ccl4 groups had the same weight to begin with. We did not actively select which mouse received what treatment but only factored in the weight to ensure a fair comparison between the two treatment arms. For the ageing experiments, mice were not randomized, but separated by age group.<br>Cell culture experiments did not require normalization because the tests were compared to controls. Plates needed to be marked to ensure the treatments were delivered to the appropriate plates (and not the control) and randomisation would not be practical or feasible.
Blinding	Investigators were not blinded during the experimental phase of the in vivo experiments (for drug administration purposes), but they were blinded for data collection. Mouse samples were stained and analysed together with no regard to group provenance in a blinded fashion. All samples were blinded as part of the analysis procedure. For the senescence prediction assessment, the person was not aware of the sample being analysed, as samples were renamed for the analysis. Investigators were not blinded during the cell culture experiments, as the investigators needed to know the groups to provide adequate treatment (against the control).

Reporting for specific materials, systems and methods

We require information from authors about some types of materials, experimental systems and methods used in many studies. Here, indicate whether each material, system or method listed is relevant to your study. If you are not sure if a list item applies to your research, read the appropriate section before selecting a response.

Materials & experimental systems

n/a	Involved in the study	
☐	☒	Antibodies
☐	☒	Eukaryotic cell lines
☒	☐	Palaeontology and archaeology
☐	☒	Animals and other organisms
☒	☐	Clinical data
☒	☐	Dual use research of concern
☒	☐	Plants

Methods

n/a	Involved in the study	
☒	☐	ChIP-seq
☒	☐	Flow cytometry
☒	☐	MRI-based neuroimaging

Antibodies

Antibodies used	The following antibodies were used for the immunofluorescent and immunohistochemistry experiments: The following antibodies were used for the immunofluorescent and immunohistochemistry experiments: mouse monoclonal anti-bromodeoxyuridine (BrdU) (3D4; BD Biosciences, 555627) 1:2000; rabbit polyclonal anti-53BP1 antibody (Novus Biologicals, NB100-304) 1:1000; goat polyclonal anti-uPAR (Novus Biologicals, AF534), 1:200; mouse monoclonal anti-Nras (Santa Cruz, sc-31), 1:500; rabbit monoclonal anti-LINE-1 ORF1p (Abcam, ab216324) 1:500; mouse monoclonal anti-phospho-Histone H2A.X (Ser139) (Sigma-Aldrich, 05-636) 1:250; rabbit polyclonal anti-p21 (2947S; Cell Signaling) 1:2000; mouse monoclonal anti-p53 (DO-1, Santa Cruz, sc-126) 1:100; rabbit monoclonal anti-p21 (EPR18021, Abcam) 1:700; rabbit recombinant monoclonal anti-GFP antibody [EPR14104] (ab183734) 1:500;<br><br>We used the following secondary antibodies: AlexaFluor 488/594 conjugated, Thermo Fisher Scientific, A11029/A11032) goat anti-mouse IgG (H + L), 1:2000; AlexaFluor 488/594 conjugated, Thermo Fisher Scientific, A11034A11037) goat anti-rabbit IgG (H + L), 1:2000; AlexaFluor 488/594 conjugated, Thermo Fisher Scientific, A11055/A11058) donkey anti-goat IgG (H+L), 1:2000.
Validation	BrdU<br>https://www.abcam.com/brdu-antibody-bu175-icr1-proliferation-marker-ab6326.html<br><br>p21<br>https://www.scbt.com/p/p21-antibody-m-19<br><br>p53<br>https://www.scbt.com/p/p53-antibody-do-1<br><br>phospho-Histone H2A.X<br>https://www.sigmaaldrich.com/ES/en/product/mm/05636i<br><br>GFP<br>https://www.abcam.com/gfp-antibody-epr14104-ab183734.html<br><br>53BP1<br>https://www.novusbio.com/products/53bp1-antibody_nb100-304?gclid=Cj0KCQjwtO-kBhDIARIsAL6LorckptuOfEvkCtvN5OgoVHacLvs5urXLuZ7WQAwLXgrrYqI1LIdZwV4aAiJsEALw_wcB&gclsrc=aw.ds<br><br>uPAR

https://www.novusbio.com/products/upar-antibody_af534

Nras
https://www.scbt.com/p/n-ras-antibody-f155?productCanUrl=n-ras-antibody-f155&_requestid=4095230

LINE-1 ORF1p
https://www.abcam.com/products/primary-antibodies/line-1-orf1p-antibody-epr21844-108-ab216324.html

Eukaryotic cell lines

Policy information about cell lines and Sex and Gender in Research

Cell line source(s)	Both female (IMR90, MCF7, HEK-293T) and male (A549, SK-HEP-1, HCT116, MRC-5) cell lines were used in this study. A549 (CCL-185), HCT116 (CCL-247), HEK-293T (CRL-11268), SK-HEP-1 (HTB-52), SK-MEL-103 (HTB-70), MRC-5 (CCL-171) and MCF7 (HTB-22) cells were obtained from the American Type Culture Collection (ATCC). Early passage IMR90 cells (ATCC CCL-186) were obtained from Coriell Institute.
Authentication	HCT116, MCF7, IMR90, SK-HEP-1 and A549 cells have been recently authenticated by DNA (STR) profile performed by EuroFins. The rest of the cell lines were not authenticated for this study.
Mycoplasma contamination	All cell lines were routinely tested for mycoplasma contamination and were consistently negative.
Commonly misidentified lines (See ICLAC register)	None of the cell lines used in this study is present in the database of commonly misidentified cell lines.

Animals and other research organisms

Policy information about studies involving animals; ARRIVE guidelines recommended for reporting animal research, and Sex and Gender in Research

Laboratory animals	Mice were kept on a 12-hour light/dark cycle and between 21-23°C temperature and 45-65% humidity levels under specific pathogen-free barrier conditions within individually ventilated cages with ad libitum access to standard chow food (SDS RM1/3 [E] LBS Serving Biotechnology) and water. C57BL6/J littermate mice were used. Animal walfare was monitored and euthanasia practices performed according to the requirements of aforementioned practice licenses and regulatory frameworks.<br>For liver fibrosis experiments 8 week year old male C57BL6/J were used.<br>For aging experiments male C57BL6/J littermates were used, with a 90-day old young cohort and a 600 day old aged cohort.<br>for liver cancer initiation and senolysis experiments, 5-6 week old female C57BL/6J were used.
Wild animals	No wild animals were used in this study.
Reporting on sex	For the ageing experiment, male C57BL/6J littlermates were utilized. For the liver cancer initiation and senolysis experiments female C57BL/6J were used. For the fibrosis experiment, male mice were used. As sex is not a factor in the scope of the study design, we acquired both male and female mice for different experiments depending on availability. Our classifiers identify senescence in samples from male and female
Field-collected samples	No field-collected samples were used in this study.
Ethics oversight	Mouse liver fibrosis experiments were performed in accordance with German Law and with the approval of the Regierungspräsidium Karlsruhe (G139/19). Aging experiments were performed in accordance to the UK Animals (Scientific Procedures) Act 1986 and amended regulations (2012) and approved by the Imperial College´s animal welfare and ethical review body (PPL 70/8700). Liver cancer initiation and senolysis experiments were performed in accordance to the UK Animals (Scientific Procedures) Act 1986 and amended regulations (2012) and approved by the Imperial College´s animal welfare and ethical review body (PPL 7009080).

Note that full information on the approval of the study protocol must also be provided in the manuscript.

Plants

Seed stocks	n/a
Novel plant genotypes	n/a
Authentication	n/a
